# A Cochlear Implant Performance Prognostic Test Based on Electrical Field Interactions Evaluated by eABR (Electrical Auditory Brainstem Responses)

**DOI:** 10.1371/journal.pone.0155008

**Published:** 2016-05-05

**Authors:** Nicolas Guevara, Michel Hoen, Eric Truy, Stéphane Gallego

**Affiliations:** 1 University Head and Neck Institute, CHU de Nice, 31 Avenue de Valombrose, 06107 Nice cedex 2, France; 2 Oticon Medical, Clinical and Scientific Research Department, 2720 chemin St Bernard, 06220 Vallauris, France; 3 Department of Audiology and Otorhinolaryngology, Edouard Herriot Hospital, 5 Place d'Arsonval, 69437 Lyon, France; 4 Institute for Readaptation Sciences and Techniques, 8 Avenue Rockefeller, 69373 Lyon Cedex 08, France; University of Salamanca- Institute for Neuroscience of Castille and Leon and Medical School, SPAIN

## Abstract

**Background:**

Cochlear implants (CIs) are neural prostheses that have been used routinely in the clinic over the past 25 years. They allow children who were born profoundly deaf, as well as adults affected by hearing loss for whom conventional hearing aids are insufficient, to attain a functional level of hearing. The “modern” CI (i.e., a multi-electrode implant using sequential coding strategies) has yielded good speech comprehension outcomes (recognition level for monosyllabic words about 50% to 60%, and sentence comprehension close to 90%). These good average results however hide a very important interindividual variability as scores in a given patients’ population often vary from 5 to 95% in comparable testing conditions. Our aim was to develop a prognostic model for patients with unilateral CI. A novel method of objectively measuring electrical and neuronal interactions using electrical auditory brainstem responses (eABRs) is proposed.

**Methods and Findings:**

The method consists of two measurements: 1) eABR measurements with stimulation by a single electrode at 70% of the dynamic range (four electrodes distributed within the cochlea were tested), followed by a summation of these four eABRs; 2) Measurement of a single eABR with stimulation from all four electrodes at 70% of the dynamic range. A comparison of the eABRs obtained by these two measurements, defined as the monaural interaction component (MIC), indicated electrical and neural interactions between the stimulation channels.

Speech recognition performance without lip reading was measured for each patient using a logatome test (64 "vowel-consonant-vowel"; VCV; by forced choice of 1 out of 16). eABRs were measured in 16 CI patients (CIs with 20 electrodes, Digisonic SP; Oticon Medical ®, Vallauris, France).

Significant correlations were found between speech recognition performance and the ratio of the amplitude of the V wave of the eABRs obtained with the two measurements (Pearson's linear regression model, parametric correlation: r2 = 0.26, p<0.05).

**Conclusions:**

This prognostic model allowed a substantial amount of the interindividual variance in speech recognition scores to be explained. The present study used measurements of electrical and neuronal interactions by eABR to assess patients' bio-electric capacity to use multiple information channels supplied by the implant. This type of prognostic information may be valuable in several ways. On the patient level, it allows customizing of individual treatments.

ClinicalTrials.gov Identifier: NCT01805167

## Introduction

Cochlear implants (CI) represent a *bona fide* 20^th^ century medical revolution. Used in clinical practice since the 1990s, this technology has allowed functional hearing to be restored in individuals with severe to profound hearing loss [1,2]. It has furthermore transformed the lives of many born-deaf children, providing them access to the world of oral communication and allowing them to develop intellectual and educational abilities often comparable to those of children with normal hearing [3]. CIs function by delivering direct electrical stimulations to the auditory nerve (cranial nerve VIII) using multi-electrode arrays that are surgically implanted in the cochlea. The “modern” CI (i.e., a multi-electrode implant using sequential coding strategies) [1] has yielded good speech comprehension outcomes. The recognition level for monosyllabic words is approximately 50% to 60%, and sentence comprehension is close to 90% (after 12 month use, without lip reading) [4]. These good average results however hide a very important interindividual variability as scores in a given patients’ population often vary from 5 to 95% in comparable testing conditions. In their 2004 study for example, Firszt and colleagues observed perception scores ranging from approximately 2 to 87% (average of 42%) for monosyllabic words in quiet [5]. This large interindividual variability can certainly be explained by many different factors identified at several levels of the auditory and speech processing chain. Identified candidates range among others, from developmental factors or age at implantation [6,7], to the quality of the electrode-neuron interface obtained after implantation [8], up to cognitive [9] or psycho-social aspects [10]. Improving our understanding of these factors and developing new, individualized approaches for using this neural prosthesis is mandatory to reduce outcome variability and optimize individual benefits. In this context, some parameters of the electrical stimulation delivered through CIs such as the number of stimulating electrodes, the speed or the frequency of stimulation, and the type of electrical stimulation used, can somewhat be customized for each patient. In practice however, customization often remains limited, due to difficulties in understanding and diagnosing the integration of the signal at the individual level.

The existence of electrical interactions between individual channels inside the cochlea [11,12] could explain important amounts variation in CI outcomes. Moreover if interactions could be assessed more efficiently, this could lead to interesting individual fine-tuning options. These interactions are responsible for an important loss in efficiency of CIs as systems today offer electrode carriers with up to 22 electrodes, when it was shown that individual patients in the end often benefit from only reduced numbers of independent spectral channels, down to four to eight [13, 14]. Although this could have a limited impact on speech recognition in silent backgrounds [15], it could become a crucial issue for the perception of speech in noise [13,16] or music [17]. The potential origins of channel interactions can be manifold and their effects could be of two opposite consequences, often difficult to disentangle. Residual polarization effects will have facilitating effects on consecutive stimulations, while refractory effects will tend to diminish subsequent neural responses (see [18] for a recent review on interaction mechanisms). CI channel interactions were studied since the introduction of the multichannel CI. Early studies quantified channel interactions using psychophysical methods evaluating loudness summation or forward masking effects across channels [19–21]. Results from these works were at the origin of the introduction of sequential stimulation strategies, avoiding to stimulate spatially adjacent electrodes at the same time. This decreased the strong interactions created by simultaneous stimulation of electrodes observed in early versions of multichannel CIs [1]. More recent studies systematically characterized channel interactions using the forward masking psychophysical paradigm in different CI models [22] or the spatial spread of excitation obtained using different pulse shapes or stimulation modes [23–27]. Even if they happen to be powerful research tools for comparing spatial spreading induced by different pulse shapes, grounding schemes or stimulation modes, such psychophysical methods for evaluating channel interactions are difficult to apply in clinical settings. These approaches are often very time-consuming and rely on the feedback of patients, which cannot always be obtained in a detailed and reliable fashion. Moreover, psychophysical methods as spatial tuning curves were shown to be relatively poor predictors of speech perception outcomes in CI [28,29], although some studies reported significant correlations with speech perception scores, see for example [30].

Faster, more reproducible and potentially more clinically-relevant evaluations may be obtained using electrophysiological evaluations of channel interactions. Early studies demonstrated the feasibility of evaluating the spatial spread of neural excitation using electrically evoked compound action potentials (ECAPs) and showed that this measure could provide robust predictions of perceptual dimensions (C and T-levels) in CI users [31]. Further experiments also suggested that an ECAP derived channel separation index could be correlated with pitch ranking [32]. ECAPs were further used to study different stimulation strategies in CIs [33–35] or to obtain detailed spectral resolution maps along electrode arrays [36]. To our knowledge, few works directly correlated ECAP derived interaction measures with speech perception outcomes. Won and colleagues (2014) reported evidence that vowel perception could be predicted by the global spectral resolution of entire electrode arrays estimated through ECAP measures but did not observe any effect at the single-electrode level [37]. This is limiting potential clinical applications as efficiently limiting channel interactions requires to modify parameters of individual electrodes. ECAPs thus provided up to now limited predictions of speech perception outcomes.

Because they constitute very peripheral measures, ECAPs may provide a good estimation of the quality of local electrode-neuron interfaces but can only poorly predict more integrated perceptual mechanisms reflecting activity in further structures of the auditory system, beyond the cochlea. Along this line, two recent papers reported evidence that combining peripheral measures via ECAPs to more central measures using the acoustic change complex (ACC) could provide more reliable estimates of spectral selectivity, significantly correlated with speech perception [38,39]. The contribution of central auditory pathways can be studied electrophysiologically by auditory evoked potentials, with an early component (known as the electrically auditory brainstem response–eABR) and a late component (corresponding to the late cortical responses). We have previously shown that the relationship between electrophysiological and speech recognition variables is more pronounced when early (brainstem) rather than late (cortical) evoked responses are considered [40–42].

To date, no quantitative method based on eABRs evaluating electrical interactions was described. Former studies reported the measurement of higher-level binaural interaction (i.e., at the level of the superior olivary complex, the core of the lateral lemniscus and the inferior colliculus) in patients with bilateral CI. These measures were performed by subtracting the eABR obtained after bilateral stimulation from the sum of the two eABRs obtained in a monaural fashion [43]. The objective was to measure the interaction between the right side and the left side. This methodology, called the *binaural interaction component* (BIC), has seen renewed success given the number of patients with bilateral implants. Nonetheless, due to the length of the tests and the lack of real clinical usefulness, this technique is rarely applied outside of research programs [44]. The aim of the current experiment was to test a novel eABR-based method of measuring interactions and to correlate the results of this testing with speech recognition performances of CI patients. Similar to the BIC, our measure is based on a comparison of eABRs, although it is obtained using a single implant. It assesses the electrical and neural interactions occurring with unilateral stimulation. By analogy, we therefore defined this test as the monaural interaction component (MIC).

## Materials and Methods

### Participants

Sixteen adults (11 female; aged between 19 and 81 years) who had undergone CI surgery (Digisonic SP; Oticon Medical, Vallauris, France) participated in this study. All were native speakers of French with post-lingual deafness onset and had used the implant for at least 10 months prior to the experiment. No specific inclusion criteria were defined, particularly in terms of etiology or duration of deafness and the time since implantation. This approach provided us access to a wide range of word recognition performances. Demographic data are presented in Table 1. This study and all experimental procedures were approved by the Institutional Review Board at the University Hospital in Nice. Each participant provided written consent and the study and all experimental procedures were approved by the South Mediterranean V Ethical Review Board (Comité de Protection des Personnes Sud Méditérrannée V, authorization n°12,075).

**Table 1:** Subject demographics.

Subject identification	Gender	Age (years)	Onset of Hearing Loss (years)	Duration of CI Use (years)	Etiology of Hearing Loss	VCV test in QuietScore (%)	IndividualPresentationLevel (dB SPL)
10-CM	F	48	40	5	Congenital	12.5	75
2-LR	M	59	22	11	Otosclerosis	18.7	75
13-CG	F	81	50	12	Unknown	18.7	70
14-GG	M	62	46	0.85	Otosclerosis	21.9	70
12-TD	F	61	44	3	Unknown	23.4	70
11-ME	F	67	43	6	Unknown	26.6	75
5-NF	F	55	52	5	Congenital	28.1	70
6-MJ	M	58	40	1.5	Congenital	37.5	65
16-GA	F	19	19	12	Congenital	45.3	70
15-DG	M	46	9	1.2	Otosclerosis	53.1	70
7-YS	F	38	35	4	Congenital	54.7	75
3-DM	F	65	26	7	Unknown	56.2	70
1-ML	F	48	11	5	Unknown	64.1	65
4-RJ	F	55	20	4	Otosclerosis	70.3	65
9-GS	F	49	20	2.5	Unknown	73.4	65
8-SP	M	52	8	1.5	Unknown	75.0	75

### The Digisonic SP CI system

The Digisonic SP is a transcutaneous multi-electrode CI system that uses the main peak interleaved sampling (MPIS) coding strategy [45]. The implanted part of the device is composed of a receptor/transducer and an electrode array that is 25 mm long, with 20 active platinum-iridium electrodes. The stimulation in this system employs a hybrid grounding scheme resulting from the simultaneous use of two types of ground electrodes. One ground electrode is positioned outside of the cochlea, as in a typical monopolar scheme, and all non-active electrodes on the electrode array are placed into a ground mode, as in a common-ground configuration. This leads to the coexistence of two possible current return paths, one intra- and one extra-cochlear. The balance between the two current return paths is modulated by their respective impedances. The monopolar pathway is favored by high impedances. As a result, the intra-cochlear pathway will be favored only for electrodes very close to the stimulation electrode. Another particularity of this system is related to the shape of the electrical pulse used for stimulation. In this device, electrical pulses have a single active phase (anodic), the current equalization being realized passively by capacitive coupling. At the time of the experiment, all patients wore the same sound processor, incorporating a post-spectral decomposition logarithmic output compression function [46].

### Measurement of speech recognition performance without lip reading

Speech recognition was assessed using a vowel-consonant-vowel (VCV), 16-alternatives forced-choice logatome test ran in quiet. VCVs were extracted from standardized lists recorded on a CD (Listes de logatomes de Dodelé, Collège National d'Audioprothèse, Paris, France) and embedded in an ad-hoc developed Matlab® (The MathWorks, Natick, Mass., United-States) program. Only consonant comprehension was tested, to both increase the difficulty of the test and increase the matching between the speech perception evaluation and the frequency areas tested by the eABR (from 1,107 Hz to 4,883 Hz). All patients were tested four times on each 16 stimuli, with a random selection, for a total of 64 trials. Stimuli were delivered through a loudspeaker placed approximately one meter in front of the patient. This test was performed in a sound treated room, at a comfortable sound level that was individually determined for each patient in order to account for the important interindividual variability in dynamic range observed in CI patients. Keeping clinical mapping settings constant, and fixing the output level to an arbitrary value could have led to very different percepts in different CI patients, equalization of listening comfort was therefore preferred. Presentation levels ranged from 65 dB SPL to 75 dB SPL (detailed in Table 1). A 1/16 comprehension level (6.25%) corresponded to random recognition.

### Electrical Auditory Brainstem Response (eABR) measurements

EABRs were recorded using surface electrodes that were placed on the forehead (+), the lower part of the chin (-), and the contralateral mastoid (common). Each testing session lasted three to four hours, including breaks. During the recordings, participants were encouraged to relax during the recording sessions to limit artifacts due to muscle activity.

The full-scale range used for eABR recording was set to 100 μV using the ABR recording system Centor USB (Racia-Alvar®). Responses were filtered using a wideband (1.6–3.6 kHz) analog bandpass filter. The averaging involved 1,500 sweeps. To estimate and confirm reproducibility, eABRs were recorded twice, reported data correspond to the first recording obtained for each participant. The recording duration was 10 milliseconds per sweep. The stimuli used to record the eABRs were generated by a PC using a portable Neurelec-Digistim® stimulator that was attached through a USB port. Stimuli were asymmetric charge-balanced biphasic pulses (phase amplitude: 0.9 mA, total pulse duration: from 20 μs to 250 μs, as a function of loudness perception, pulse-rate: 47 Hz, pulse onset asynchrony adjusted depending on pulse duration). Stimuli were delivered to four different electrodes distributed along the cochlea (electrode numbers: 4, 7, 10, and 13, from base to apex) at a unique, individually adjusted stimulation level. Tested electrodes were selected because they correlated with the primary area required for word comprehension. Indeed the articulation index tells us that the frequencies between 1 and 3 kHz contribute most to speech intelligibility [47]. Electrodes 4, 7, 10, and 13 are situated approximately at 4.6, 8.2, 11.8, and 15.4 mm from the base of the cochlea (estimated from the electrode-array length of 25 mm; electrode length: 0.5 mm; inter-electrode distance: 0.7 mm). In acoustic terms these electrodes code for the frequency bands: 4,120–4,883 Hz, 2,409–2,799 Hz, 1,497–1,758 Hz, and 1,107–1,237 Hz, respectively (default parameters of frequency-mapping in the Digisonic SP CI system). Given that the threshold (T) and maximal comfort (M) levels for the electrical stimulation can vary considerably across individual patients and electrodes, these levels were specified as a percentage of the electrical dynamic range of the tested electrode, rather than an absolute intensity. For each electrode tested, we calculated the difference between T- and M- Levels and set the stimulation level to the 70% value of this electrical dynamic range. This ensured robust and reliable eABR recordings, maximizing the chance of obtaining well-defined wave-V responses, without the risk of reaching the discomfort threshold during the stimulation of the four electrodes in a same-pulse-train.

Two types of measurements were performed: i) **Individual stimulations** were obtained by recording four different eABRs from each single electrode among the four test electrodes (4, 7, 10, and 13). The sum of these four eABRs was subsequently calculated. ii) **Multi-electrode stimulations** were obtained by recording one eABR trace using a sequential pulse- train stimulation from the same four electrodes. Using the MPIS strategy, the delay between the first pulse occurring at electrode number 4 and the last pulse of the pulse train at electrode number 13 is 84 μs.

### eABR analyses and assessment of the monaural interaction component (MIC)

For each participant, the eABR recordings obtained from the four individual stimulations were summed, leading to one eABR trace per participant in this recording condition and one second eABR trace in the multi-electrode stimulation condition. We then derived the MIC, mathematically defined as the ratio between the peak amplitudes of the wave-V components obtained from the summed individual and multi-electrode stimulations. The amplitude peak of wave-V was selected both for a practical and theoretical issue. In practice, wave-V is more robustly observed in patients than wave-III, which can be sometimes harder to identify. In theory, wave-V is generated higher in the auditory system (inferior colliculus) than wave-III is (superior olivary complex) and may represent a better candidate to observe a correlation with integrated auditory processes such as speech perception. Several works could indeed report that the inferior colliculus would represent sparse speech cues [48,49].

We hypothesized that the MIC would represent an approximation of the interaction between the concerned electrodes:

- In the theoretical scenario where there would be no electrical and/or neural interactions, we would assume that the neural response triggered by the stimulation at each individual electrode would be completely independent from each other in both recording modes, individual or multi-electrode. In this case, the sum of the four eABRs obtained by individual stimulations should be very similar to the eABR obtained with the multi-electrode stimulation.
- On the opposite, in the theoretical case where electrical and/or neural interactions would be total, the eABR obtained from the multi-electrode stimulation would actually tend to be equal to the eABR obtained from a pseudo-monoelectrode implant. Upon multi-electrode stimulation, the eABR obtained would tend to be equal to the eABR obtained from a single electrode. The sum of the four eABRs obtained from individual stimulations should then be four times larger than the eABR obtained from the multi-electrode stimulation.

## Results

**Fig 1:**
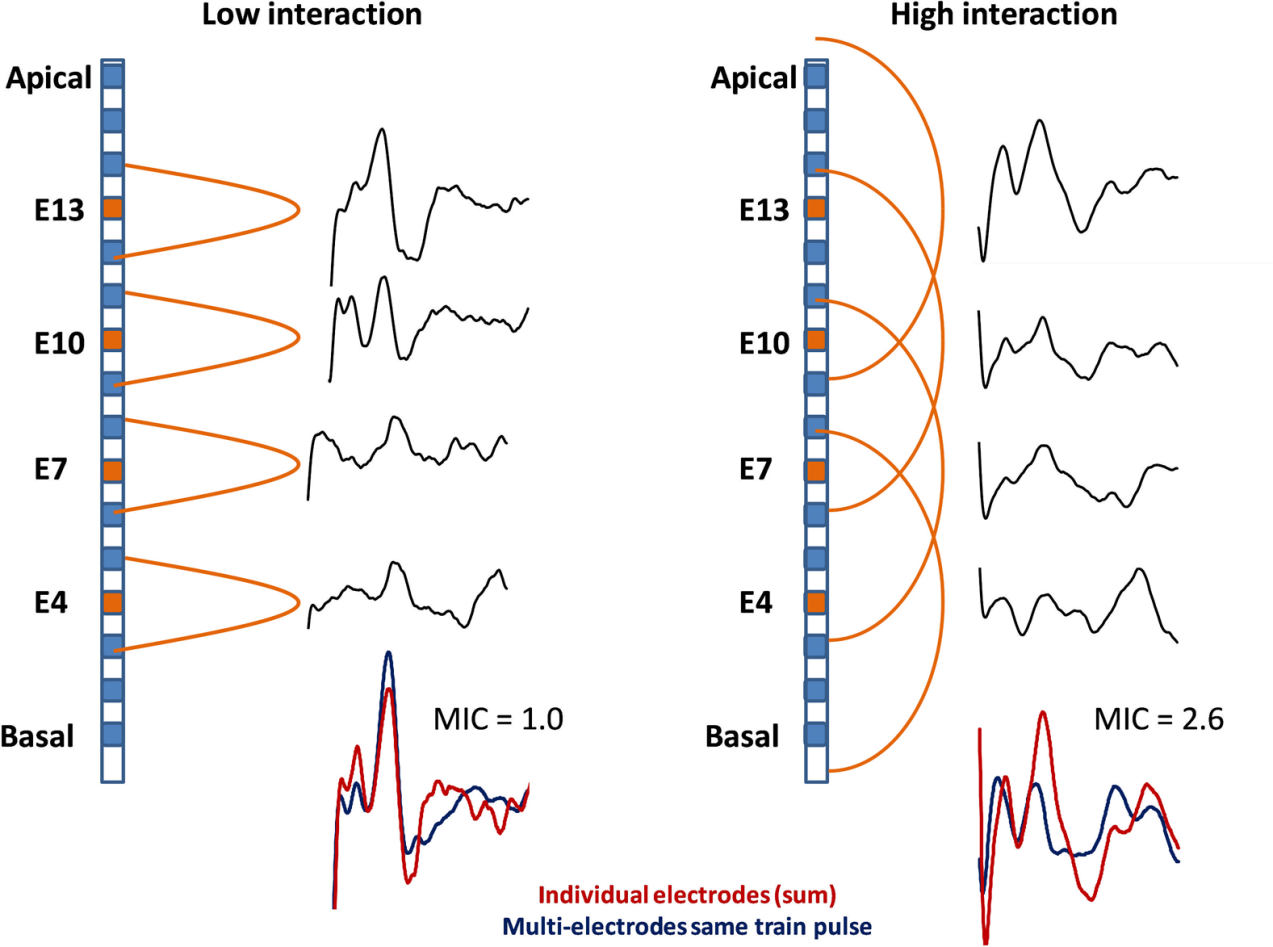
Schematic representation of the electrical interaction assessment method by eABR. Left: low-interaction case. The sum of the four eABR measures obtained from individual stimulations (red) equals the eABR obtained with the multi-electrode stimulation (blue), the MIC value tends to 1. Right: high-interaction case: the eABR amplitude obtained from the multi-electrode stimulation tends to equal that of one eABR measure in the individual recording, the MIC tends to rise and constitutes a metric of the electrical interaction.

Good quality eABRs were obtained in most cases, with a clear wave V, while wave III could only be identified in 13/16 cases. To better explain the protocol and serve as an example, Fig 1 shows and illustration of the low- (top-left) and high (top-right) interaction cases and the eABR recordings obtained from two individual patients using our method. Fig 1 bottom-left, the patient appears to show little interaction. The sum of the eABRs for individual stimulations was similar to the multi-electrode stimulation eABR and the observed MIC was close to 1. On the right side of Fig 1, the other patient showed a higher level of cross-channel interaction. The sum of the eABRs for individual stimulations was much higher than that of the multi-electrode stimulation eABR, leading to a MIC value of 2.6. Stimulation of the same four electrodes in the same pulse-train generated an important overlap. These first observations suggested that the MIC could be a good indicator of channel interaction in CI patients.

**Fig 2:**
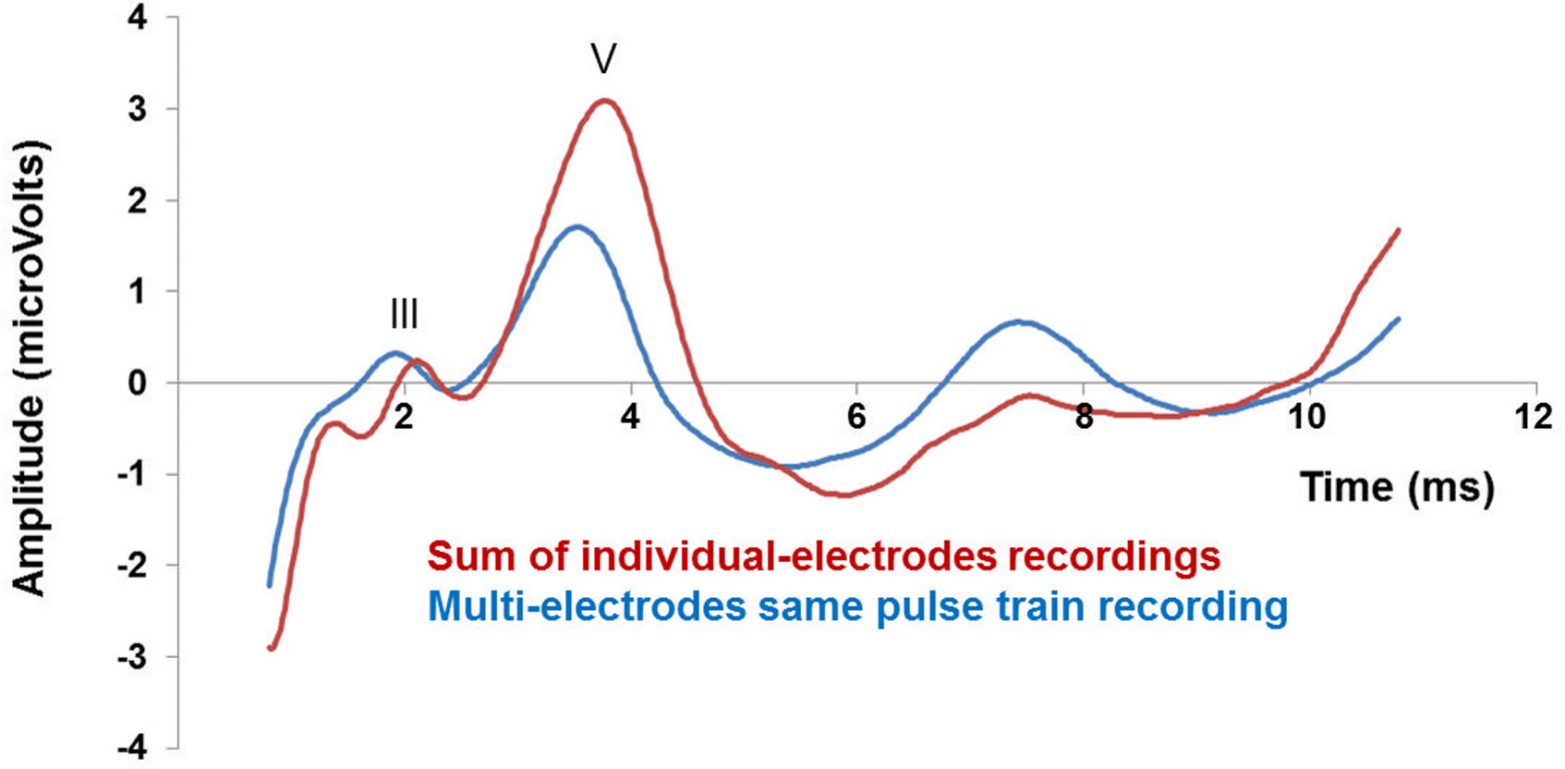
Grand-averaged eABRs obtained in the multi- and individual-electrode conditions. Red: grand-averaged eABR (N = 16) obtained by summing the traces from the individual-electrodes recordings. Blue: grand-averaged eABR (N = 16) obtained from the same pulse-train, multi-electrodes stimulation. III: peak of wave-III. V: peak of wave-V.

We then analyzed the main characteristics of waves III and V for the 16 patients, these observations are summarized in Table 2. eABR traces obtained for the multi- and individual-electrode recordings were averaged across participants in order to generate grand-average traces. As can be seen on Fig 2, the grand-average eABR reveals differences between the components obtained from the sum of individual-electrodes stimulations and from the multi-electrode stimulations. The first main difference concerned latencies of wave-III and wave-V components, which both appeared earlier in the multi-electrode condition. Both peak-latencies were significantly different, respectively: 1.93 ms ^+^/_-_ 0.18 *vs*. 2.08 ms ^+^/_-_ 0.12, difference = 0.154 ms (t (12) = -2.90, p = 0.013) for wave-III and 3.63 ms ^+^/_-_ 21 *vs*. 3.83 ms ^+^/_-_ 0.21, difference = 0.200 ms (t (15) = -3.56, p = 0.003) for wave-V. As expected, wave III could only be characterized precisely in 13 out of the 16 included participants, the amplitude analyses were therefore restricted to wave-V. The amplitudes of wave-V observed for both recording conditions were also significantly different, the average amplitude being lower in the multi-electrode condition: 3.01 μV ^+^/_-_ 0.18 than in the individual electrode condition 4.41 μV ^+^/_-_ 0.26 (t (15) = -3.48, p = 0.003). These results show that, on average, the multi-electrode stimulation does not generate an ABR response equivalent to the sum of four individual stimulations, presumably reflecting the effect of across channel interactions. These interactions are characterized by a shorter latency and diminished amplitude in the multi-electrode stimulation condition.

**Table 2:** Speech and Electrophysiological outcomes.

Participant Id.	Latency wave III (ms)		Latency wave V (ms)		Amplitude wave V (nV)		MIC	VCV score (%)
	Multi	Individual sum	Multi	Individual sum	Multi	Individual sum		
10-CM	nd	nd	3.62	3.98	198.08	671.80	3.39	12.5
13-CG	2.18	2.18	3.36	3.60	34.52	94.28	2.73	18.7
2-LR	nd	nd	3.86	3.82	470.01	930.75	1.98	18.7
14-GG	2.02	2.38	3.92	3.90	177.99	420.88	2.36	21.9
12-TD	2.20	1.98	3.82	3.90	378.48	382.06	1.01	23.4
11-ME	nd	nd	3.88	4.14	45.78	68.54	1.50	26.6
5-NF	1.70	1.88	3.38	3.72	433.96	359.97	0.83	28.1
6-MJ	1.70	2.12	3.62	3.96	271.10	418.30	1.54	37.5
16-GA	1.86	1.96	3.48	3.52	418.27	540.63	1.29	45.3
15-DG	1.98	2.16	3.68	4.24	218.56	228.90	1.05	53.1
7-YS	1.68	2.04	3.32	3.74	314.72	497.90	1.58	54.7
3-DM	1.74	2.12	3.42	3.80	802.84	1003.50	1.25	56.2
1-ML	1.94	2.00	3.46	3.48	365.59	352.42	0.96	64.1
4-RJ	2.02	2.16	3.94	3.60	165.82	209.93	1.27	70.3
9-GS	1.92	2.04	3.60	3.86	349.99	636.09	1.82	73.4
8-SP	2.16	2.08	3.76	4.08	168.36	243.67	1.45	75.0
N	13	13	16	16	16	16	16	16
Average	1.93	2.08	3.63	3.83	300.88	441.23	1.63	42.47
STD	0.18	0.12	0.21	0.21	182.01	259.30	0.67	20.94
T-Tests		**0.01336**		**0.00287**		**0.00336**		

**Fig 3:**
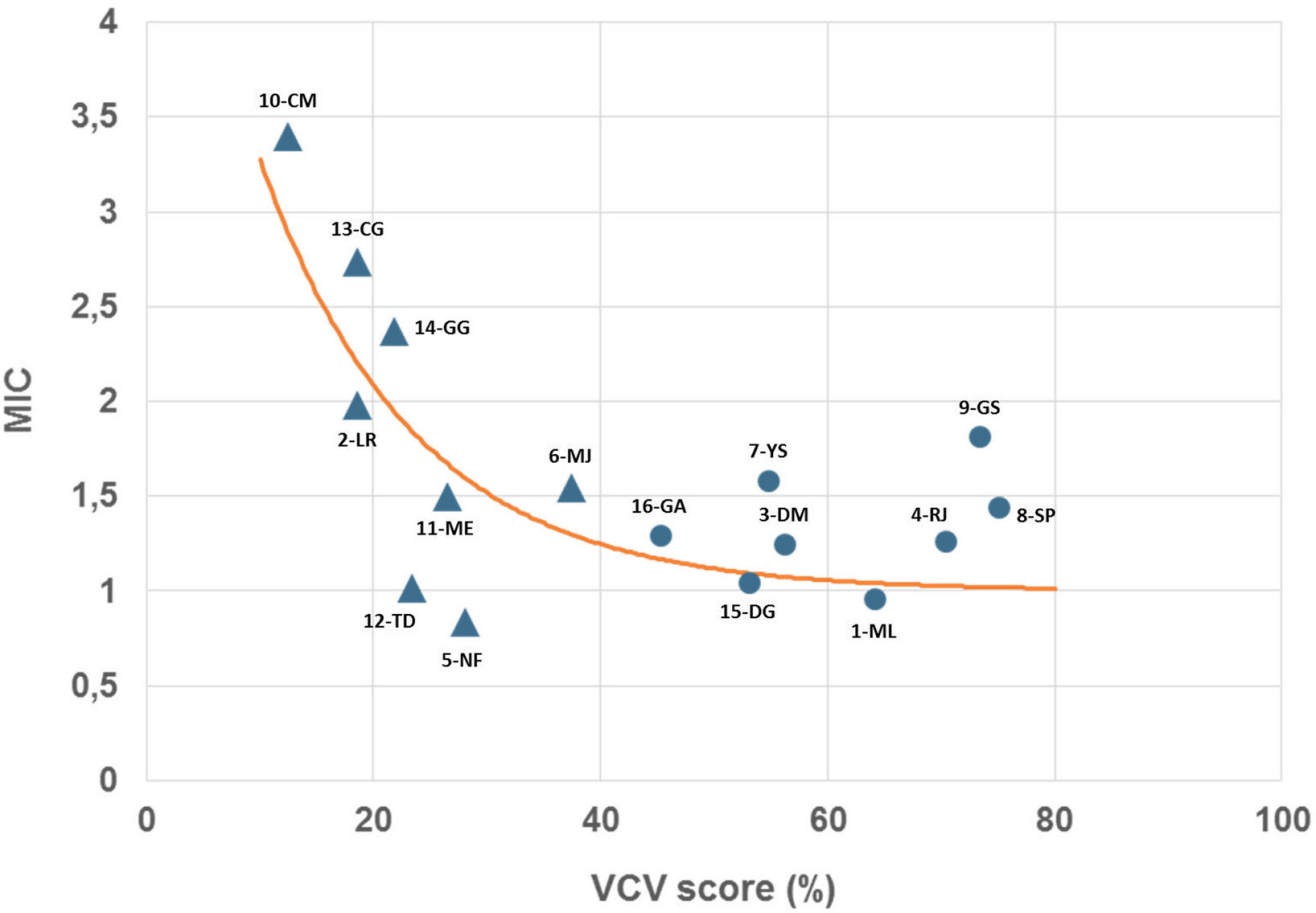
Prognostic model of the speech recognition performance as a function of the electrical interaction measured by EABR. Individually calculated MIC values compared to VCV scores. Triangles: the eight low speech performing patients (VCV score < 40%). Circles the eight best performing patients (VCV scores > 40%). Orange curve: the decreasing exponential non-linear regression matching the data and following equation: *MIC* = 1 + 2.8*e*^−0.04(*VCVscore*−6.25)^.

In order to further characterize the effects of interactions as evaluated by our ABR recording paradigm, we calculated differences between the two recording conditions (sum of individual vs. multi-electrode) for each participant for the latencies of wave-III and wave-V. For the amplitudes, we quantified by the MIC calculated as the ratio of wave-V amplitudes. Pearson’s parametric correlations were used to test for a relationship between VCV scores and these different parameters. The results of this analysis are displayed in Table 3. All factors led to non-significant correlations with speech scores, except for MIC (n = 16, r^2^ = 0.26, p<0.05). Data reflecting the relationship between individual MIC values and VCV perception scores are shown in Fig 3. In order to evaluate if a simple mathematical model could better fit data, we modeled the relationship using a decay exponential with the following criteria: i) MIC values constrained between 4: maximal interaction and 1: no interaction; and a chance-level of 6.25% (1/16) in the VCV perception test. A decreasing exponential non-linear regression was performed with Microsoft® Excel 2000/XLSTAT^©^-Pro (Version 4.07, 2013, Addinsoft, Inc., Brooklyn, NY, USA), which matched the model Eq (1):

$$MIC=1+{2.8e}^{-0.04(VCVscore-6.25)}$$ (1)

In (1), MIC being the MIC value and VCV score the percentage of correct responses obtained with the speech perception test. The resulting function was mapped on the individual data as shown in Fig 3. The fact that the data are well explained by an exponential decay points to an interesting property of our dataset. As the speech score increases from low to medium values, (in our experiment for participants 10-CM with 12.5% to 6-MJ with 37.5% recognition score (triangles in Fig 3) the MIC value decreases exponentially from high values around 3.5 to around 1.5. This is suggesting that the participants with very low speech score (<40% VCV recognition score closed-set in quiet) in general have high MIC values while patients with higher speech recognition score (>47.5% in our sample) have low MIC values. This difference remained however non-significant in our study as reflected by a non-significant p-value for a two independent heteroscedastic samples t-test (t(14) = 1.80, p = 0.09). Given the size of the two compared sample (n = 8) this observation still suggests that MIC values rapidly decrease with the augmentation of speech perception scores.

**Table 3:** Difference analyses and correlations with speech-scores.

Participant Id.	Latency differencewave III(ms)	Latency differencewave V(ms)	MIC	VCV score (%)
10-CM	nd	0.36	3.39	12.5
13-CG	0.00	0.24	2.73	18.7
2-LR	nd	-0.04	1.98	18.7
14-GG	0.36	-0.02	2.36	21.9
12-TD	-0.22	0.08	1.01	23.4
11-ME	nd	0.26	1.50	26.6
5-NF	0.18	0.34	0.83	28.1
6-MJ	0.42	0.34	1.54	37.5
16-GA	0.10	0.04	1.29	45.3
15-DG	0.18	0.56	1.05	53.1
7-YS	0.36	0.42	1.58	54.7
3-DM	0.38	0.38	1.25	56.2
1-ML	0.06	0.02	0.96	64.1
4-RJ	0.14	-0.34	1.27	70.3
9-GS	0.12	0.26	1.82	73.4
8-SP	-0.08	0.32	1.45	75.0
N	13	16	16	16
Average	0.15	0.20	1.63	42.47
STD	0.18	0.22	0.67	20.94
Correlation with VCV score				
R^2^	0.000	0.042	0.255 *	-
p-value	.974	.940	.046 *	-

*: Significant correlations with speech scores

Finally, and in order to evaluate the effect of the possible confounding factor of the level of presentation used in the VCV test, which was individually adjusted for each subject in order to account for individual variations in comfort levels across CI patients, we also calculated the correlation between scores at the VCV test expressed in % and the presentation level expressed in dB SPL. This correlation remained non-significant (n = 16, r^2^ = 0.16, p = 0.12). Finally, both factors were entered into a multiple-regression analysis in order to estimate their respective contributions to speech perception scores. This observation suggested that the VCV scores obtained in the present experiment were not significantly influenced by the individually adjusted comfortable level of presentation of the VCV stimuli.

## Discussion

The substantial variability in functional results with CIs, combined with a lack of reliable prognostic factors, limit the individual optimization of this neural prosthesis. We have developed a prognostic test that uses eABRs to measure global electrical and neural interactions at the level of the electrode array. It assesses the biological capacity of the patients to use several information channels supplied by the implant and can explain a substantial amount of the interindividual variability found in speech perception scores among CI patients. We also observed that the speech perception data were well explained by an exponential decrease of the electrical interactions as evaluated by the MIC. Given the number of participants included in the present study it was however not possible to reach significance when studying the difference between low- and high- speech proficient participants, this limit of the current project suggests the interest of running a large multicentric study involving a larger population of CI patients in order to confirm and extend the present observations. Another limitation of the present approach concerns the fact that, for the clinical relevance of the approach, participants were tested using a clinical processor in clinical settings, imposing some compromises. For example, we decided to keep the mapping parameters of the patients and run the tests with these parameters, thereby preventing us from harmonizing the level of presentation of the speech stimuli across participants and choosing to ask participants to indicate a comfortable level of presentation. The range of presentation levels was in the end relatively restricted, between 65 and 75 dB SPL, but this could have introduced a bias in the speech perception measures. Further studies will be dedicated to reproducing the present observations with a larger population to confirm the present observations. A caveat associated to the MIC approach as introduced in the present article is associated to the simultaneous measure of ABR responses from multiple electrodes. In the hypothetical case where the amplitude of wave V on one of the electrodes would completely dominate both the sum of the single electrode measures as well as the multi-electrode measure, we would observe a small MIC not necessarily reflecting low channel interactions. Future developments will therefore concentrate on developing measures based in individual electrode measures. Nevertheless, despite these compromises, we were able to identify a significant relationship between an eABR derived estimation of channel interactions, the MIC, and speech perception outcomes in CI users. Moreover, the MIC measure showed some interesting distribution properties. Patients with high MIC values had poor speech perception levels, suggesting that high electrical interactions are detrimental to speech perception. However, low electrical interactions as evaluated by the MIC seem to be a necessary but not sufficient condition to observe medium to high speech perception scores, as participants with low MIC values >1.5, showed a large range of speech perception scores, approximately from 23 to 75%. This result suggests that other dimensions also play a crucial role in observing good speech perception outcomes with CIs, the number of independent spectral channels is important but other factors come into play. Numerous predictive factors for post-CI outcomes were described in former studies, including large multicentric analyses of prognostic factors in adults, performed in 1996 and updated in 2013 [50,51]. These analyses confirmed the importance of the following four main factors: the duration of severe deafness prior to placement of the CI, the age at onset of severe deafness, the etiology of the deafness, and the duration of CI use. In 1996, a model defined by these four factors accounted for 21% of interindividual variance in CI outcomes. However, with the widening of CI indications, and improvements in signal processing allowing always more participant to reach higher perception scores, the overall CI performance improved as did the variance of outcomes, causing these initial prognostic factors to explain only 10% of the variance in 2003. Despite the introduction of nine new significant factors in this predictive model (including the duration of moderate hearing loss and the use of hearing aids), only 22% of the variance could ultimately be explained. These disparities in post-CI outcomes are likely influenced strongly by the physiology of the auditory nerve, as well as auditory pathways in the brainstem and auditory cortex of the wearer of the implant, up to the influence of cognitive factors. These components are highly variable in terms of functional integrity and capacity to adapt (functional plasticity), this variability may at least partly explain the differences in results that persist to date among individuals who have received CIs.

Our results strongly suggest that, at least for patients with poor speech perception, a significant amount of their speech perception variability can be explained by cross-channel electrical interactions.

Channel interactions can be measured using different approaches, including electrical field imaging (EFI), evaluating interactions at the physical level, ECAPs, allowing an estimation of physiological interactions and psychophysics measures evaluating the effect of interactions at the perceptual level, see [18] or [36] for reviews. In 2011, Tang, Benitez and Zeng have explored interactions at the three levels, combining the three approaches in the same CI patients. Comparing the interaction estimation obtained at the three levels revealed that interindividual variability increased from physical measures with EFI to physiological and behavioral measures, suggesting that higher-order interactions as revealed by ECAPs or behavioral measures were better reflecting individual variability in CI outcomes than electrical field imaging, and that reducing these higher-order interactions could be helpful to reduce variability in CI outcomes. In the present work, we introduced a novel approach to evaluating higher-order interactions by using an electrophysiological measure (amplitude of the eABR wave-V component), which we believed would be closer to behavioral variability as physical EFI or ECAP measurements would be. Due to the complexity of recording eABRs, the majority of electrophysiological studies regarding electrode-neuron interactions have used ECAP measurements, which are fast and easy to perform. However, ECAP measurements have several limitations. This measurement of the auditory nerve response to electrical stimulation is recorded by another intracochlear electrode. It therefore only reflects the cochlear nerve response (and only a part of it). Additionally, ECAP is affected by its close interface with the intracochlear recording electrode. eABRs instead allow the measurement of the progressive auditory brainstem elaboration of the auditory information, leading to complex percepts as speech for example. In particular, using the amplitude wave-V component reveals interactions observed at the level of the inferior colliculus, a neural relay that was shown to contain elaborated auditory representations relevant to speech perception [40–42, 40, 49]. As a result, if ECAP measures can be used to predict low-level percepts (thresholds or comfort levels) [31], their application to predict speech outcomes was up to now limited [37]. As suggested by the results from the present study, eABRs may reflect speech perception scores more accurately. Further studies will be dedicated to extend this observation to the individual electrode level in order to improve the clinical relevance of the approach.

Four different neural interaction phenomena were described, which may be characterized as temporal, that participate to cross-channel interactions in CI: refractoriness, facilitation, accommodation and spike-rate adaptation [18]. The two latter ones are mostly related to temporal integration factors which are certainly minored in the paradigm used in the present experiment using a low-rate stimulation (47Hz) and a sequential stimulation on distant electrodes. The first two ones could however be observed, especially in the multi-electrode same pulse-train stimulation condition. The fact that this condition was associated to a significant decrease in the latency of the wave-III and V components suggests a facilitating effect due to residual polarization consecutive to the short delay pulses. A decrease in latency of the eABR components is usually associated with an increase in the amplitude of the stimulation [52]. However, the fact that this decrease in latency was in our study accompanied by a decrease in amplitude relative to the sum of the wave-V amplitude observed with individual recordings suggests that our method also identified interactions due to refractory effects between channels. The reduced number of usable spectral channels is due to diffusion of the signal from the stimulated electrodes and the resultant interactions with the overlapping neural population. ECAP measurements allow observation of these overlaps [34], but they do not allow an analysis of how these peripheral interactions are integrated and processed in the first auditory relays. In addition, there is variability among the electrode-neuron interface within a cochlea, and these variations probably explain the lack of correlation between ECAPs and performance as measured by a VCV test, which is related to the treatment of multiple electrodes activation throughout the cochlea. Auditory performance can therefore be predicted in part by the characteristics of the eABRs. Nonetheless, due to the complexity of the neural processes involved speech processing, many other factors (including more central ones) might contribute to the variability in CI outcomes.

### Outlook of the methods

Our eABR-based assessment of interactions could be used to develop novel ways of improving CI mapping in a more individualized way. It is likely that an actual decrease in neuronal interactions allows optimizing the central coding of auditory information provided by the implant. Nonetheless, before identifying ways to decrease the interactions, methods to measure interactions that correlate with speech recognition performance are necessary.

For any given patient, interactions beyond the cochlea (and the electrode-neuron interface) are linked to intrinsic factors (e.g., the number of residual spiral ganglion cells and the temporal synchrony of neuronal discharges). Currently, there is no easy way to directly modulate the nervous system (e.g., with the use of growth factors). However, it may be possible to act on information coding strategies sent by the implant. Techniques to focus and direct the electrical current using different modes of stimulation (“current steering and current focusing”) have been proposed to reduce its diffusion [53]. Tripolar stimulations were shown to improve speech in noise perception compared to monopolar stimulations [54] (but see [55] for inconsistent results) and current focusing can provide better spectral resolution [56,57]. However, other limitations (e.g., power consumption related to threshold increase and the use of multiple current sources), as well as difficulties in adapting proper programming to the right patient have limited the routine use of these stimulation modes. Another option would be to alter the algorithm for selecting the “N-of-M” peaks (peak-picking algorithm); deactivating specific channels according to the measured interactions could also potentially be of benefit, as already shown by Noble and colleagues using a CT-derived imaging method [58] or as implemented in the PACE/MP3000 strategy, which selects channels based on psychoacoustic simultaneous masking trying to minimize channel interaction [59]. Lastly, decreasing the stimulation frequency for patients in whom many interactions have been measured might help to limit these interactions. Although a decrease in transmitted information may result, speech recognition performance might also improve.
